# First epidemiological characterization of *Leishmania tarentolae* in sand flies collected in southern France and Corsica Island

**DOI:** 10.1371/journal.pntd.0014526

**Published:** 2026-07-20

**Authors:** Stefania Porcelli, Julie Sevila, Nalia Mekarnia, Anne-Laure Baňuls, Pascal Delaunay, Aurélien Mercier, Bruno Mathieu, Stéphane Goldblum, Arezki Izri, Loïc Favennec, Fano Randrianambinintsoa, Jérome Depaquit, Jorian Prudhomme, Florence Robert-Gangneux

**Affiliations:** 1 Univ Rennes, Inserm, EHESP, Irset (Institut de Recherche en Santé Environnement Travail), UMR_S 1085, Rennes, France; 2 Unité des Virus Emergents (UVE: Aix-Marseille Univ, Università di Corsica, IRD 190, Inserm 1207, IRBA), Marseille, France; 3 UR ESCAPE - USC Anses PETARD, SFR Cap Santé, University of Reims Champagne-Ardenne, Reims, France; 4 UMR MIVEGEC (University of Montpellier-IRD-CNRS), LMI DRISA, Montpellier, France; 5 Service de Parasitologie Mycologie, CHU Nice, CS 23079, Nice, France; 6 Inserm U1094, IRD UMR270, Univ. Limoges, CHU Limoges, EpiMaCT - Epidemiology of Chronic Diseases in Tropical Zone, Institute of Epidemiology and Tropical Neurology, OmegaHealth, Limoges, France; 7 Institute of Bacteriology and Parasitology, UR 3073 PHAVI, Medical Faculty, University of Strasbourg, Strasbourg, France; 8 Laboratoire de Parasitologie, UR ESCAPE, Université de Rouen Normandie, Rouen, France; 9 Laboratoire de Parasitologie-Mycologie, CNR Cryptosporidioses, Microsporidies et Autres Protozooses Digestives, Centre Hospitalier Universitaire de Rouen, Rouen, France; 10 Laboratoire de Parasitologie-Mycologie, Centre Hospitalo-Universitaire, pôle de Biologie territoriale, Reims, France; 11 Laboratoire de Parasitologie-Mycologie, CHU Rennes, Rennes, France; University of Bremen: Universitat Bremen, GERMANY

## Abstract

*Leishmania tarentolae*, a reptile-associated parasite, is poorly documented in France. This study provides the first molecular survey of *L. tarentolae* in sand flies across mainland France and Corsica (2023–2024), combining morphological and molecular vector identification with PCR-based parasite detection. A total of 2,731 female sand flies were collected, and 412 unfed pools and 132 blood-fed females were analysed. *Sergentomyia minuta* was the main carrier, with higher infection rates than *Phlebotomus perniciosus*. Among unfed females, *L. tarentolae* was detected in 56 pools, mainly from Corsica, whereas only two positive pools were found in mainland France. One pool from Montpellier in mainland France was positive for *L. infantum*. Of the 132 blood-fed females analysed, 24 tested positive for *L. tarentolae*. Blood-meal analyses revealed multi-host feeding, humans, livestock, hares and first evidence of goat feeding in *Se. minuta*, indicating flexible feeding behaviour. Co-circulation of *L. tarentolae* and *L. infantum* complicates diagnostics and epidemiology. These findings expand the known range of *Sauroleishmania* in Western Europe and provide a framework for entomological and eco-epidemiological surveillance. Integrated monitoring of vectors, reptiles, and domestic animals is essential for assessing transmission risks amid environmental and climatic changes.

## Author summary

Leishmaniasis is a parasitic disease transmitted by sand flies and caused by different *Leishmania* species. In southern France, *Leishmania infantum* infects dogs and humans and is a well-known public health concern. Another species, *Leishmania tarentolae*, is mainly associated with reptiles such as geckos and is not considered pathogenic to humans. However, because these parasites may circulate in the same areas and share sand fly species, their coexistence could complicate disease surveillance and diagnosis.

We carried out the first nationwide molecular survey of *L. tarentolae* in sand flies collected in mainland France and Corsica over two transmission seasons. We found that this parasite is widely present in Corsica and also occurs in southern mainland France. It was most frequently detected in *Sergentomyia minuta*, a sand fly species commonly associated with reptiles, but it was also found in other sand fly species. Infected sand flies had fed on humans and domestic animals, showing that contact between reptile-associated parasites and mammals occurs in natural settings.

These findings show that multiple *Leishmania* species co-circulate in Mediterranean France. Surveillance programs should consider this diversity to ensure accurate diagnosis and better assessment of transmission risks in a changing environmental context.

## Introduction

*Leishmania* (*L*.) protozoan parasites are maintained in complex zoonotic cycles involving phlebotomine sand flies and a wide range of vertebrate hosts across the Mediterranean basin, including the southern regions of France [1]. While considerable attention has focused on pathogenic species such as *Leishmania infantum*, increasing evidence indicates that the subgenus *Sauroleishmania*, classically associated with reptiles, circulates within the same eco-epidemiological landscapes [2]. Among these reptile-associated species, *L.* (*Sauroleishmania*) *tarentolae* has emerged as the most extensively studied, owing to its genetic proximity to mammal-infective *Leishmania* and its suitability as an experimental model. First described by Wenyon in 1921, *L. tarentolae* is naturally maintained in geckos of the genus *Tarentola*, particularly *T. mauritanica*, which represents its principal vertebrate reservoir [3].

Although *L. tarentolae* has long been regarded as non-pathogenic to mammals, recent reports of its DNA or evidence of transient infections in dogs, combined with experimental observations of its interactions with mammalian cells, have rekindled interest in its ecology, host range, and potential implications for leishmaniasis surveillance in areas where it coexists with *L. infantum* [2,4–6]. These findings challenge long-standing assumptions regarding the strict reptile specificity of *Sauroleishmania* and highlight the need for updated epidemiological assessments in Mediterranean foci.

In southern France, the earliest ecological investigations conducted in the Pyrénées-Orientales region provided the first evidence of *L. tarentolae* infection in *T. mauritanica* geckos [3]. These studies also documented the presence of abundant populations of *Sergentomyia minuta*, a predominantly herpetophilic sand fly. Subsequent detections of promastigotes in wild-caught *Se. minuta*, exhibiting heterozygous phosphoglucose isomerase enzymatic profiles clustering with *L. tarentolae* isolates, further supported the involvement of this species in the local transmission cycle. Experimental infections conducted earlier by Parrot (1935) demonstrated intense midgut multiplication of *L. tarentolae* in *Se. minuta*, followed by expulsion of parasites after digestion without anterior migration, a pattern consistent with ingestion-based rather than bite-based transmission [7]. Collectively, these studies contributed to a Mediterranean framework, largely shaped by the Maazoun-Rioux school, in which *Se. minuta* is considered the primary vector responsible for sustaining the saurian *L. tarentolae* cycle [3,8]. However, the absence of recent field investigations in France greatly limits contemporary understanding of its distribution, infection dynamics, and ecological interactions.

Across the central and western Mediterranean basin, *L. tarentolae* has been reported in *T. mauritanica*, sympatric sand fly species, and occasionally in domestic animals, suggesting complex multi-host interactions that overlap with human leishmaniasis foci [2]. Molecular surveys from neighbouring countries, including Spain and Portugal [9,10], have confirmed the presence of *L. tarentolae* DNA in sand fly vectors. Yet, the most extensive and detailed molecular data concerning its circulation in reptiles, dogs, and sand flies remain concentrated in Italy. There, a multi-site survey detected *L. tarentolae* DNA in about 10% of tested Squamata reptiles (26/259), particularly *Podarcis siculus* and *T. mauritanica* captured in Apulia, Calabria, and Sicily [2]. In a dog-shelter setting in Apulia, *L. tarentolae* was identified in 35.7% of *Podarcis siculus* lizards (10/28), while duplex qPCR screening of 294 female sand flies revealed the parasite in both *Se. minuta* (21/231; 9.1%) and *Phlebotumus perniciosus* (2/52; 3.8%) [2,11]. These findings collectively indicate that *L. tarentolae* may circulate across multiple phlebotomine species in southern Italy.

In France, seven sand fly species (*Ph. ariasi*, *Ph. mascittii*, *Ph. papatasi*, *Ph. perfiliewi*, *Ph. perniciosus*, *Ph. sergenti*, and *Se. minuta*) have been historically recorded on the continent, with five also reported in Corsica (*Ph. mascittii*, *Ph. perniciosus*, *Ph. sergenti*, and *Se. minuta*) [12]. Within this assemblage, *Se. minuta* stands out as the most plausible vector supporting saurian *Leishmania* transmission in southeastern France.

This study aimed to screen for *Leishmania* spp. across multiple French regions and to assess the distribution of *L. infantum* and *L. tarentolae*. This objective was pursued through sand fly surveillance activities conducted within the Climate Monitoring and Decision Support Framework for Sand Fly-borne Diseases (CLIMOS project, https://climos-project.eu/), a Horizon Europe initiative supporting preparedness against climate-driven vector-borne diseases.

## Results

### Sand fly fauna

A total of 2,731 female sand flies were collected over two trapping seasons (2023–2024), with 1,235 (45%) captured in mainland France and 1,496 (54%) in Corsica. Sand flies were detected from mid-June to mid-October with higher numbers of specimens trapped in July and August (Table 1), although trapping effort was not standardised across all months.

**Table 1:** Monthly number of collected female sand flies in different sites during the study period (2023-2024).

	April	May	June	July	August	September	October	November
Balagne	0	0	0	2	254	0	0	0
Corte	0	0	117	561	243	203	15	0
Sartène	0	0	0	28	0	0	0	0
Conca	0	0	0	0	73	0	0	0
Montpellier	0	0	51	400	646	31	0	0
Strasbourg	0	0	1	0	0	0	0	0
Saint-Ythaire	0	0	0	9	8	0	0	0
Toulouse	0	0	0	5	0	0	0	0
Surba	0	0	0	65	9	0	0	0
Limoges	0	0	0	2	0	0	0	0
Nice	0	0	0	4	4	0	0	0
Rennes	0	0	0	0	0	0	0	0
Rouen	0	0	0	0	0	0	0	0
Reims	0	0	0	0	0	0	0	0
Paris	0	0	0	0	0	0	0	0
Total	2,731							

They were mainly captured in Southern France and Corsica, but a few specimens were trapped in Strasbourg and Saint-Ythaire, and, for the first time, in Limoges. No sand flies were captured around Rennes, Rouen, Reims, and Paris. Overall, 2,599 and 132 females were unfed and fed, respectively (Tables 1–2).

**Table 2:** Number of female sand flies by species trapped in mainland France and Corsica,2023-2024.

Species	Mainland France No. SF (% of trapped SF)	Corsica Island No. SF (% of trapped SF)	No. unfed pools	No. blood-fed females
*Sergentomyia minuta*	22 (1.7%)	617 (41.2%)	135	28
*Phlebotomus ariasi*	1,140 (92.3%)	0	81	74
*Phlebotomus perniciosus*	36 (2.9%)	828 (55.4%)	155	29
*Phlebotomus mascittii*	9 (0.7%)	17 (1.1%)	17	0
*Phlebotomus sergenti*	0	22 (1.5%)	21	1
Unidentified	28 (2.2%)	12 (0.8%)	10	0
Total	1,235 (45%)	1,496 (54%)	419	132

The majority of sand flies collected in southern France, were identified as *Ph. ariasi* (92.9%), whereas in Corsica *Ph. perniciosus* and *Se. minuta* were co-dominant species, representing 55.4% and 41.2% of trapped sand flies, respectively. The number of trapped sand flies by species is depicted in Table 2.

The 2,599 unfed females were grouped into 412 pools containing 1–30 individuals, consisting of 135 *Se. minuta* (33%), 155 *Ph. perniciosus* (37.6%), 81 *Ph. ariasi* (19.6%), 17 *Ph. mascittii* (4.12%), and 14 *Ph. sergenti* (3.4%) pools. Ten pools could not be identified to species level and were classified as unidentified. The 132 blood-fed females were identified as 74 *Ph. perniciosus* (56%), 29 *Ph. ariasi* (21%), 28 *Se*. *minuta* (21%), and one *Ph. sergenti* (0.75%) (Table 2).

### Molecular screening and identification of *Leishmania* spp. in unfed sand flies

Among the 412 unfed pools analysed, 25 tested positive by kDNA qPCR with a Ct ≤ 40 (mean Ct = 37.7 ± 3.6), and six tested positive considering a Ct threshold < 38 (mean Ct = 33 ± 5.2) (Table 3). One (Ct = 23.3) of these 25 kDNA-positive pools, consisting of 28 *Ph. ariasi* sand flies collected in 2024 in Montpellier (mainland France), was identified by *hsp70* sequencing as *L. infantum* (GenBank accession number PX943776). In addition, *hsp70* sequencing identified 12 pools and seven fed females as being positive for *L. tarentolae* in Corsica and Montpellier (Genbank accession numbers: PX943768, PX943769, PX943770, PX943771, PX943772, PX943773, PX943774, PX943775, PX943777, PX943778, PX943779, PX943780, PX993421, PX993422, PX993423, PX993424, PX993425, PX993426, PX993427. Following these initial identifications, all unfed pools were further screened using the *L. tarentolae* specific PCR to better quantify the circulation of this *Leishmania* species. Fifty-four pools collected in Corsica and two pools from mainland France tested positive for *L. tarentolae* PCR (mean Ct = 28.6 ± 6.9) (Tables 3–4). Nine of the 25 kDNA-positive pools were also positive for the *L. tarentolae* specific PCR. *L. tarentolae* PCR-positive samples showed a kDNA mean Ct of 38 ± 2. Only two unfed-female pools from mainland France tested positive for *L. tarentolae* (Tables 3–4).

**Table 3:** Results of kDNA and *L. tarentolae* qPCRs screening.

Sand fly pools with positive detection by PCR or *hsp70* sequencing	*L. tarentolae* qPCR N positive (Ct mean ± SD)	kDNA qPCR (Ct ≤ 40) N positive (Ct mean ± SD)	kDNA qPCR (Ct < 38) N positive (Ct mean ± SD)
Total number (n = 57)	50 (28.7 ± 7)	25 (37.7 ± 3.6)	6 (33 ± 5.2)
*Ph. perniciosus* (n = 13) ^a^	9 (34.8 ± 6.2)	8 (38 ± 3)	1 (31.2)
*Se. minuta* (n = 42) ^^b^^	41 (27 ± 6.3)	9 (37.7 ± 2)	3 (35.2 ± 0.6)
*Ph. sergenti* (n = 4)	Nd	4 (39.2 ± 0.5)	Nd
*Ph. ariasi* (n = 2) ^c^	36	2 (30.6 ± 10)	2 (30.6 ± 10.3)
*Ph. mascittii* (n = 1)	Nd	1 (39.6)	Nd
Unidentified (n = 1)	Nd	1 (39.6)	Nd

^a^Two pool were positives with the two qPCR.

^b^Six pools were positive with the two qPCR.

^c^One pool was positive with the two qPCR.

Nd: not detected.

**Table 4:** Unfed females sand flies tested for *Leishmania* spp. in mainland France and Corsica Island in 2023 and 2024 by qPCR and/or *hsp70* sequencing.

Year	No. of trapped SF	No of pools analyzed	No. of pools positive for *Leishmania infantum*		No. of pools positive for *Leishmania tarentolae*	
			Corsica Island	Mainland France ^a^	Corsica Island ^b^	Mainland France ^c^
2023	868	176	0	0	25	2
2024	1731	236	0	1 ^^d^^	29	0
Total	2,599	412	0	1	54	2

SF: sand fly;

^a^identified with *hsp70* sequencing.

^b^four identified with *hsp70* sequencing, 50 positives with *L. tarentolae* PCR (of which seven were identified by *hsp70* sequencing).

^c^one positive with *L. tarentolae* PCR and one identified with *hsp70* sequencing.

^d^SF species: *Phlebotomus ariasi.*

**Fig 1:**
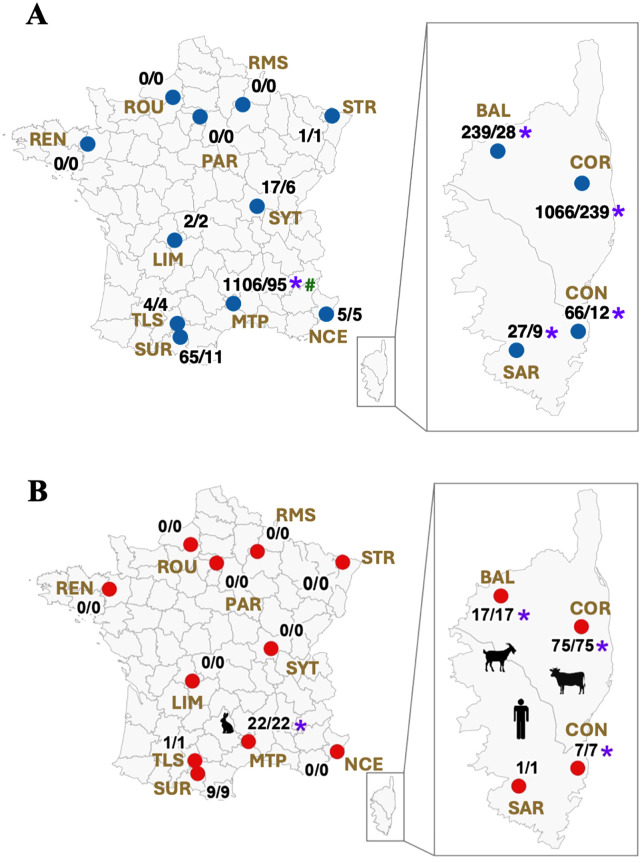
Maps showing trapping sites in mainland France and Corsica. Locations where *Leishmania tarentolae* was detected are indicated by an asterisk (*), while the location where *Leishmania infantum* was detected is marked with a hash (#). **(A)** Unfed females. Numbers indicate the total number of sand flies collected and the number of analysed pools. **(B)** Blood-fed females, with blood meal sources identified by icons. The maps were created in R using the maps package (CRAN: https://cran.r-project.org/web/packages/maps/index.html). Basemap data were obtained from UNESCO (1987) via UNEP/GRID-Geneva. Montpellier: MTP, Nice: NCE, Toulouse: TLS, Limoges: LIM, Rennes: REN, Rouen: ROU, Reims: RMS, Strasbourg: STR, Saint-Ythaire: SYT, Paris: PAR, Surba: SUR, Balagne: BAL, Corte: COR, Sartène: SAR, Conca: CON.

Overall, of the six kDNA PCR-positive pools with Ct < 38, only one from Montpellier was confirmed to be infected with *L. infantum*, while the five remaining pools were actually infected with *L. tarentolae.* Besides, *L. tarentolae* was identified in several sites in Corsica (Balagne, Corte, Sartène, Conca) and in Montpellier in mainland France (Fig 1).

Among the 56 pools positive for *L. tarentolae*, 13 and 42 were issued from *Ph. perniciosus* and *Se. minuta* pools, respectively, and one from *Ph. ariasi*. The mean Ct for *L. tarentolae* detection was significantly lower in *Se. minuta* than in *Ph. perniciosus* (27 ± 6.3 *vs* 34.9 ± 6.2, respectively, *p* < 0.01) (Table 3).

Additionally, *Se. minuta* supported significantly higher levels of *L. tarentolae* (by *L. tarentolae*-specific qPCR and/or *hsp70* sequencing) circulation than *Ph. perniciosus*. Specifically, 42 pools of the 611 *Se. minuta* individuals tested positive, corresponding to a MIR of 7% (95% CI: 5.0–9.1), whereas 13 pools of the 787 *Ph. perniciosus* individuals tested positive, corresponding to a MIR of 1.65% (95% CI: 0.8–2.8). The difference between *Se. minuta* and *Ph. perniciosus* was statistically significant (Fisher’s exact test, *p* < 0.0001). One *Ph. ariasi* pool was positive only by *hsp70* sequencing and negative by *L. tarentolae*-specific qPCR, corresponding to a MIR of 0.09%. No positive pools were detected in *Ph. mascittii* and *Ph. sergenti*. Finally, the ten unidentified pools tested negative for *L. tarentolae* (Table 5).

**Table 5:** *L. tarentolae* detection in unfed sand fly pools during 2023–2024, according to species.

SF species	No. tested pools	No. positive pools ^a^	MIR (%)	95%CI	*p*-value
Unfed pooled females					<0.001
*Sergentomyia minuta*	135	42	7%	5.0-9.1%	
*Phlebotomus perniciosus*	155	13	1.65%	0.9-2.8%	
*Phlebotomus ariasi*	81	1	0.09%	N/A	
*Phlebotomus mascittii*	17	0	N/A	N/A	
*Phlebotomus sergenti*	14	0	N/A	N/A	
Unidentified	10	0	N/A	N/A	
Total	412	56			

SF, sand fly; MIR, minimal infection rate; CI, confidence interval; N/A: not available.

a No. positive pools include pools that tested positive with the *L. tarentolae*‑specific qPCR or by *hsp70* sequencing (in one pool of *Se. minuta*, four pools of *Ph. perniciosus* and 1 pool of *Ph. ariasi, L. tarentolae* was identified only by *hsp70* sequencing).

b Ct values are shown only for *L. tarentolae*‑specific qPCR‑positive pools.

**Fig 2:**
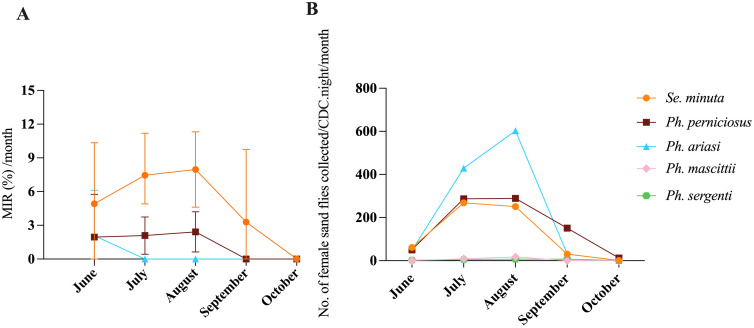
Minimum infection rate (MIR) of unfed female sand flies positive for *Leishmania tarentolae* in Corsica Island during 2023 and 2024. **(A)** Monthly variation in MIR for *Se. minuta, Ph. perniciosus*, and *Ph. ariasi*; error bars indicate 95% confidence intervals. **(B)** Monthly density of female sand flies collected during the study period, expressed as number of females per CDC/night.

The *L. tarentolae* MIR values rose from June, reaching a maximum in August, at 7.9% (95% CI: 4.62–11.32) in *Se. minuta* and 2.4% (95% CI: 0.63–4.20) in *Ph. perniciosus* (Fig 2A). This pattern broadly overlapped with the monthly abundance of *S. minuta* and *Ph. perniciosus* females collected, which was highest in July-August (Fig 2B). Indeed, the peak abundances were recorded in July and August, with 268 and 251 *Se. minuta* and 287 and 289 *Ph. perniciosus* specimens being trapped over two nights, respectively (Fig 2B). In *Ph. ariasi*, MIR was 2.08% (95% CI 0.00–6.12) in June and 0% thereafter. Overall, the month with the highest MIR for the two main vector species coincided with the period of greatest sand fly abundance.

### Detection of *L. tarentolae* in fed females and blood meal analysis

Among the 132 blood-fed females, 24 tested positive for *L. tarentolae* by qPCR or *hsp70* sequencing, including 14 *Se. minuta* (50%), 7 *Ph. perniciosus* (9%) and 3 *Ph*. *ariasi* (10%) (Table 6). Both *L. tarentolae*-positive *Ph. ariasi* were trapped in the region of Montpellier (Fig 1B). One *L. tarentolae* was negative by qPCR and positive by kDNA PCR (Ct = 39.9) and sequencing (Genbank accession number: PX993422).

**Table 6:** Detection of *L. tarentolae* in blood-fed sand flies trapped in mainland France and Corsica, according to species (2023-2024).

Sand fly species	Detection rate n/N (%)	Ct qPCR *L. tarentolae*
Fed females		
*Phlebotomus perniciosus*	7/74 (9%)	33.3 ± 5.4
*Sergentomyia minuta*	14/28 (50%)	29.1 ± 9.3
*Phlebotomus ariasi* ^a^	3/29 (10%)	39.9 ± 1
*Phlebotomus sergenti*	0/1	NA

n/N: No. of positive sand fly/ total No. of sand flies.

NA: not applicable.

a 1 was negative by *L. tarentolae* qPCR and positive by *hsp70* sequencing.

Blood-meal source identification was subsequently performed on the 24 *L. tarentolae*-positive blood-fed females, all collected from rural environment. Host identification was successful in 18 specimens (75%), revealing feeding on humans (77%), goats (11%), cattle (5%), and hares (5%). At the species level, *Ph. perniciosus* fed on humans, goats, and cattle, whereas *Se. minuta* fed mainly on humans and less frequently on goats (Table 7).

**Table 7:** Identification of blood-meal sources on vertebrate hosts according to sand fly species (*n* = 24 fed females).

Blood meal source (scientific name)	Sand fly species		
	*Phlebotomus perniciosus*	*Sergentomyia minuta*	*Phlebotomus* *ariasi*
Human (*Homo sapiens*)	4*	10	
Goat (*Capra hircus*)	1	1	
Hare (*Lepus Linnaeus*)			1
Cattle (*Bos taurus*)	1		
Total (identified blood meals)	6	11	1
Total (non-identified blood meals)	2	3	1

* 1 was negative by *L. tarentolae* PCR and positive by kDNA PCR and by *hsp70* sequencing.

## Discussion

Building on the historical detections of *L. tarentolae* in geckos and *Se. minuta* from the Pyrénées-Orientales [3,7], this study represents the first comprehensive molecular survey of *L. tarentolae* in sand flies across both mainland France and Corsica. It establishes a robust reference framework for the future entomological and eco-epidemiological surveillance of this saurian-associated parasite in France, extending the known distribution of *Sauroleishmania* in Western Europe and filling a major data gap in this region, by contrast to neighbouring Mediterranean countries. Recent studies confirmed the presence and expanding distribution of *T. mauritanica*, the primary gecko host of *L. tarentolae*, across France including southern regions and Corsica-like Mediterranean habitats, with ecological niche models, forecasting northward range shifts under future climates that could facilitate parasite maintenance cycle [13,14]. This gecko’s establishment in human-modified environments like those in Provence-Alpes-Côte d’Azur supports the observed reptile-associated *L. tarentolae* circulation, highlighting its role in vector-host dynamics [13,14]. Within this context, the apparent absence of detectable *L. infantum* in Corsica fits with a scenario of low-intensity, ecologically fragmented transmission, possibly constrained by limited vector abundance, patchy suitable habitats, and insufficient large-scale screening for *L. infantum* in sand flies and reservoir hosts [15,16]. In France, recent analyses of phlebotomine sand fly distribution also indicate marked spatial heterogeneity, supporting the possibility that low-prevalence foci may remain undetected in fragmented island settings [12]. Given that Corsica lies in a marginal or emerging risk area for zoonotic leishmaniasis, this pattern is compatible with sporadic, cryptic transmission rather than true absence of the parasite, and underscores the need for expanded, species-specific molecular surveillance in future risk-mapping exercises [15,17].

Back on the mainland, the coexistence of *L. tarentolae* and *L. infantum* in the same ecological settings increases the complexity of surveillance, molecular diagnosis, and risk interpretation [1,2].

Overall, as expected, the abundance of *Se. minuta* and *Ph. perniciosus* rises from June to July, peaks in July-August, and then gradually declines to near-zero levels by October, with the most pronounced decrease occurring from September onward [18,19].

Across both survey years, *Se. minuta* was consistently the primary carrier of *L. tarentolae*, in accordance with findings reported in the literature [10,20,11]. In both years, the MIR values reflected this dominance. *Sergentomyia minuta* showed substantial infection pressure in unfed females (MIR 7%), while *Ph. perniciosus* exhibited considerably lower MIR (1.65%). Although the present study did not directly investigate reptile hosts, we collected a substantial number of *Se. minuta* specimens (*n* = 639) and its detection as the main *L. tarentolae*-positive sand fly species is consistent with previous Mediterranean studies, often in environments where reptiles occur [2,21].

Overall, *Se. minuta* sustained higher *L. tarentolae* infection pressure than *Ph. perniciosus*, as previously observed in Italy, where screening of 294 female sand flies revealed the parasite in both *Se. minuta* (21/231; 9.1%) and *Phlebotumus perniciosus* (2/52; 3.8%) [2,11]. This suggests that *Se. minuta* may have a more stable and efficient reptile-associated transmission cycle in this species, while the comparatively lower and more variable infection pressure in *Ph. perniciosus* may reflect sporadic exposure or less efficient maintenance of *L. tarentolae* in this vector. Because the host-seeking behaviour of many *Phlebotomus* species shifts with local conditions, seasonal variation, and host availability, their feeding patterns are often opportunistic [22,23]. Inter-annual differences may be thus influenced by climatic fluctuations, microhabitat humidity, temperature, or host availability, all of which are known to affect sand fly density and parasite circulation in Mediterranean transmission systems [1,21,24].

Here, blood-meal identification sources highlighted the presence of multi-host interactions. Human, goats, cattle, and hare blood meals were identified. To our knowledge, this is the first report showing that *Se. minuta* could feed on goats, apart from reptiles. Similar opportunistic feeding behaviours have been reported in other *Sergentomyia* species [25]. Although *Se. minuta* is classically considered reptile-oriented, the detection of mammalian blood meals highlights a more flexible feeding behaviour that may increase the frequency of incidental *Sauroleishmania* encounters in mammals [26–28], even if sustained mammalian transmission remains unlikely due to vector parasite compatibility constraints. Similar patterns have been reported in Italy, where *Se. minuta*, *T. mauritanica*, *L. tarentolae*, and *L. infantum* coexist in densely human-modified environments, emphasizing the need to better understand cross-host contacts and transient mammalian infections in sympatric systems [2]. Interestingly, *Se. minuta* collected at the farm fed mainly on humans, despite the fact that human presence on site was limited to only a few hours per day and no dwellings were located nearby.

The coexistence of *Sauroleishmania* with established *L. infantum* transmission, and the occurrence of *Se. minuta* feeding on humans, both add diagnostic complexity in French Mediterranean regions, especially when PCR assays are not fully discriminative at the species level. Indeed, we observed that the qPCR assay targeting *Leishmania* kDNA, which is widely used for diagnostics in humans [29], could also amplify *L. tarentolae,* though inconstantly. Therefore, in samples with low parasite loads, such as those common in field surveys, misidentification of *L. tarentolae* as a mammal-pathogenic *Leishmania* could lead to false epidemiological and diagnostic inferences. It is thus critical to apply a Ct threshold, to avoid misdiagnosis with *L. tarentolae*. In our collection, we overlooked all kDNA PCR results with Ct > 38 to increase specificity, as sand flies can harbour various trypanosomatids, which could cross-react with this qPCR [30]. Even applying this cut-off, five (mean Ct = 35) of the six positive pools, were also positive with the *L. tarentolae* qPCR (mean Ct = 25), suggesting that a Ct result >35 for the kDNA PCR should be taken with caution in geographical areas of high endemicity for *L. tarentolae*. On the other hand, the apparent poor sensitivity of the kDNA-PCR assay for *L. tarentolae* detection could explain the under recognition of *L. tarentolae* importance in France until now.

In this context, it is important to consider the complementary and partially divergent performances of the molecular tools used in our survey. Broad-range kDNA qPCR offers high analytical sensitivity for *L. infantum* and other mammal-infective species [29,31], but its amplification efficiency for *Sauroleishmania* appears reduced, as indicated by the late Ct values observed in our *L. tarentolae*-positive pools. The *L. tarentolae*-specific qPCR described by Álvaro et al. (2025) targets a *Sauroleishmania*-specific sequence and therefore combines high analytical sensitivity with species-level specificity for this parasite in sand flies, enabling the detection of additional positive pools that remained negative with the generic kDNA qPCR or *hsp70* sequencing [32].

The active circulation of *L. tarentolae* in France, and more generally in Southern Europe, raises another important concern regarding diagnosis and serological surveys. Indeed, antigen cross-reaction has been described and could be responsible for false positive reactions in serological screening studies [33]. This risk is relevant for veterinary diagnostic workflows in France, where *L. infantum* is endemic and clinical interpretation must account for background environmental *Sauroleishmania* circulation [1,21,34]. In human diagnostics, a western-blot (Western-blot *Leishmania*, LD Bio, Lyon, France) is considered as a reference test in terms of sensitivity but it can be positive in patients infected with various *Leishmania* species responsible for cutaneous leishmaniasis [35], thus the risk of cross-rection with *L. tarentolae* is likely.

Routine surveillance in Mediterranean regions should therefore incorporate molecular assays or sequencing approaches explicitly capable of distinguishing reptile-origin *Sauroleishmania*, improving case attribution in both vectors and vertebrates [34]. Priority steps include targeted sampling of geckos (*T. mauritanica*, a species present in France and already reported as infected by *L. tarentolae* [3]), expanded seasonal monitoring of *Se. minuta*, and attempts to isolate or genotype circulating *L. tarentolae* strains to assess local diversity and mammalian infectivity potential. Given the influence of climate-driven sand fly expansion in Europe, integrating entomological, reptile, and canine surveillance will support refinement of transmission risk maps, as vectors continue to colonize peri-urban corridors [1,2,21,24].

Through the integration of standardized PCR-based parasite detection, morphological and molecular sand fly species identification, and extensive field sampling, this study provides new insights in *L. tarentolae* circulation alongside *L. infantum* in French transmission foci. These results not only refine the biogeography of *Sauroleishmania* in Western Europe but also underscore the importance of considering mixed *Leishmania* communities within vectors and vertebrate hosts.

## Conclusions

This study provides, to our knowledge, the first nationwide molecular screening of *L. tarentolae* in field-caught sand flies in France, helping to fill an important national data gap and to refine current knowledge of *Sauroleishmania* distribution in Western Europe. *Sergentomyia minuta* showed the highest infection rates, although additional sand fly species were also infected, suggesting a broader eco-epidemiological involvement. Climate-driven changes affecting reptile hosts may facilitate parasite spread. Co-circulation with *L. infantum* in shared foci highlights the need for species-discriminant molecular tools and confirmatory sequencing to avoid diagnostic misclassification in sand flies, dogs, and humans. Future priorities include parasite isolation from local vectors and reptile reservoirs and experimental studies to clarify transmission potential and mammalian relevance. Surveillance systems accounting for *L. tarentolae* presence will be important for accurate risk assessment and interpretation of *Leishmania* detections in Mediterranean France.

## Materials and methods

### Sand fly collection and identification

Sand fly trapping was conducted monthly from April to November in 2023 and 2024 using CDC miniature light traps (John W. Hock Co., FL, USA).

Sampling followed standardized CLIMOS procedures: CDC miniature light traps were deployed for two consecutive nights per month from 6:00 p.m. to 8:00 a.m. at each site, yielding 1,605 trapping sessions at 15 sites (11 in mainland France: Montpellier, Nice, Toulouse, Surba, Limoges, Rennes, Rouen, Reims, Strasbourg, Saint-Ythaire and Paris), and four in Corsica (Balagne, Corte, Sartène and Conca). The traps were placed either indoors (in houses, henhouses and shelters) or outdoors (near animal shelters). The sampling effort is reported as number of sand flies/ CDC.night.

At the end of each sampling period, collected specimens were transported to the laboratory under cool conditions to preserve sample integrity as previously described [36]. The heads and genitalia were cleared in Marc-André solution and identified based on the pharynx and/or the male genitalia or female spermathecae [37–39]. Unfed specimens were pooled (≤ 30 individuals) by site, date, sex, and species and stored at -80 °C. Only female specimens were included in this study. Blood-fed females were analyzed individually for blood-meal identification.

### DNA extraction

In mainland France, sand flies were manually homogenized in 700 µL of DPBS (Dulbecco’s Phosphate-Buffered Saline). Whereas in Corsica, sand flies were homogenized using a TissueLyser (Qiagen, France) with a 3-mm tungsten bead in 700 µL of MEM (Minimum Essential Medium). Homogenates were centrifuged (14,000 rpm, 5 min, 4 °C), and were stored at −80 °C. For unfed females, DNA extraction was performed from 200 µL of the homogenate combined with 200 µL of ATL buffer (Qiagen) and 20 µL of proteinase K and incubated at 56 °C for 2 hours. DNA was then extracted using 400 µL of the prepared lysate and eluted in a final volume of 90 µL. DNA extraction was performed using the EZ1 DSP Virus Kit (Qiagen, France) on the EZ1 Advanced XL device following the manufacturer’s recommendations; this method was validated for *Leishmania* detection in sand flies and ensures high and reproducible DNA yields [40].

For blood-fed females, DNA was extracted using the MagMAX Viral/Pathogen Ultra Nucleic Acid Isolation Kit (Applied Biosystems), following the manufacturer’s instructions.

### Molecular screening of *Leishmania* infection by kDNA-qPCR

All sand fly DNA samples were first screened by a real-time quantitative PCR (qPCR) assay targeting the kinetoplast minicircle DNA (kDNA) to detect *Leishmania* spp. DNA. This initial assay was applied to all DNA extracts obtained from both unfed female pools and from individual blood-fed females. Amplification was performed on a QuantStudio5 system (QS5; Thermo Fisher Scientific, France), using kDNA-specific primers and a TaqMan probe, described by Mary et al [29]. Results with Ct values < 40 were considered positive.

### Detection of *L. tarentolae* by Real-Time PCR

All samples were additionally tested by a species-specific real-time PCR assay targeting kinetoplast minicircle DNA of *L. tarentolae* [32]. Amplification was performed using the QuantStudio 5 system, and specific primers and probe described by Alvaro et al. (2025) [32]. Each 25 μL qPCR reaction mix included 5 μL of DNA sample, 12.5 μL of TaqMan Universal Master Mix 2X and a final concentration of 625 nM of primers and 500 nM of probe. DNA was amplified using the following conditions: initial step at 95 °C for 10 min, followed by 45 cycles at 50 °C for 30 sec and 1 min at 60° C. Results with Ct values < 40 were considered positive.

### *Leishmania hsp70* sequencing

Samples that tested positive by kDNA-qPCR were then subjected to the heat-shock protein 70 (*hsp70*) sequencing for *Leishmania* species identification. The amplification and sequencing from sand fly DNA samples was performed using several primer sets, as previously described [41].

Amplified products from all reactions were purified and sequenced employing the BigDye Terminator v3.1 kit (Applied Biosystems, France) on an ABI Prism 3130XL sequencer. Multiple sequence alignments were then performed using the SeqScape software (Applied Biosystems). The aligned consensus sequences were visually inspected and edited as needed for accuracy. For species identification and confirmation, sequences were compared against reference sequences using the BLAST tool from the NCBI database and aligned with reference sequences in MEGA5 software [42], using bootstrap method 2000.

### Blood meal characterization from fed-female

Blood meals were identified in individual blood-fed female by direct sequencing of a 350 bp fragment of the cytochrome b (cyt b) gene, amplified with universal vertebrate primers, as previously described [43]. Each 50 μL PCR reaction contained 50 pmol of each primer, 10 μL of DNA template, 10 mM dNTP mix, 5 μL of 10 × buffer, and 0.25 μL of Taq polymerase (Roche Diagnostics). Amplification was performed in a thermal cycler under the following conditions: an initial denaturation at 95°C for 5 min, then 40 cycles of 30s at 95°C, 30s at 55°C, and 30s at 72°C, followed by a final extension at 72°C for 10 min. Sanger sequencing was conducted by a commercial provider (GENEWIZ, Leipzig, Germany). Sequence assembly and editing were performed using Pregap and Gap software [44,45], and the resulting sequences were compared with those in GenBank using the NCBI BLASTn tool. Blood-meal sources were assigned when the highest BLAST hit showed ≥ 98% sequence identity.

### Data analysis

The Minimum Infection Rate (MIR) was calculated following the standard approach, defined as: (number of positive pools ÷ total number of specimens tested) × 100. This metric provides a conservative estimate of parasite prevalence under the assumption that each positive pool contains at least one infected sand fly, and the values refer to a single sand fly species collected within a defined time frame and geographical area [46,47]. The MIR of *L. tarentolae*, 95% confidence interval and statistical comparison tests (Fisher’s exact test) were estimated using GraphPad v10.1 (Prism) software (Boston, Massachusetts USA). Statistical significance was set at *p* < 0.05. Spatial distribution maps were generated using R studio software (version 2024.12.0.467) [48], with the “maps” package as the basemap source. The basemap shapefile included in this package was originally obtained through UNESCO (1987) via UNEP/GRID-Geneva.
